# Short-Term Variability and Predictors of Urinary Pentachlorophenol Levels in Ohio Preschool Children

**DOI:** 10.3390/ijerph120100800

**Published:** 2015-01-14

**Authors:** Marsha Morgan, Paul Jones, Jon Sobus

**Affiliations:** United States Environmental Protection Agency, 109 T.W. Alexander Drive, Research Triangle Park, NC 27711, USA; E-Mails: jones.paul-a@epa.gov (P.J.); sobus.jon@epa.gov (J.S.)

**Keywords:** biomarkers, urine, children, determinants, phenol, home, daycare center

## Abstract

Pentachlorophenol (PCP) is a persistent and ubiquitous environmental contaminant. No published data exist on the temporal variability or important predictors of urinary PCP concentrations in young children. In this further analysis of study data, we have examined the associations between selected sociodemographic or lifestyle factors and urinary PCP concentrations in 115 preschool children over a 48-h period and assessed the 48-hour variability of urinary PCP levels in a subset of 15 children. Monitoring was performed at 115 homes and 16 daycares in Ohio (USA) in 2001. Questionnaires/diaries and spot urine samples were collected from each child. The median urinary PCP level was 0.8 ng/mL (range < 0.2–23.8 ng/mL). The intraclass correlation coefficient for urinary PCP was 0.42, which indicates fairly low reliability for a single sample over a 48-h period. In a multiple regression model, age of home and ln(creatinine levels) were significant predictors and sampling season, time spent outside, and pet ownership were marginally significant predictors of ln(urinary PCP levels), collectively explaining 29% of the variability of PCP in urine. To adequately assess short-term exposures of children to PCP, several spot urine measurements are likely needed as well as information regarding residence age, seasonality, time spent outdoors, and pet ownership.

## 1. Introduction

Pentachlorophenol (PCP) is a semi-volatile, chlorinated aromatic hydrocarbon [1]. Until the mid-1980s, PCP was commonly used as a pesticide to protect wood products from insect and fungal damage in domestic, commercial, and industrial settings in the United States (U.S.) [2,3]. It was also widely used as an antimicrobial agent in products such as food storage containers, paints, adhesives, leathers, ropes, papers, and construction materials [2,4,5]. However in 1987, the U.S. Environmental Protection Agency (U.S. EPA) cancelled almost all uses of PCP except as a wood preservative for limited industrial applications (e.g., telephone poles and railroad crossing arms) [2,3]. The U.S. EPA has classified PCP as a probable human carcinogen (Group 2B) due to its adverse health effects in exposed mammals [3,6].

PCP is a persistent and ubiquitous environment contaminant [7]. It has been detected in air, soil, carpet dust, food, and hand wipes samples collected at U.S. homes and childcare centers [8,9,10,11,12]. Previous research has indicated that dietary ingestion and inhalation are the major routes of non-occupational exposures of humans to PCP [12,13,14,15]. After absorption into the body, the lipophilic PCP is metabolized in the liver and is mainly renally eliminated as free PCP (74%) and PCP-glucuronide (12%) [16,17].

Only a few cross-sectional studies have been published on the levels of PCP in the urine of young children (<6 years old) in the U.S. [11,12,18]. Hill *et al.* [18] reported median urinary PCP levels of 14 ng/mL in 197 Arkansas (AK) children, ages 2–6 years old, in the late 1980’s. In another smaller study, Wilson *et al.* [11] estimated mean urinary PCP concentrations of 0.3 ng/mL in nine preschool children, ages 2–5 years, from North Carolina (NC) in 1997. More recently in 2000–2001, we showed median urinary PCP levels of ~0.6 ng/mL in 257 NC and Ohio (OH) preschool children, ages 2–5 years old, from the Children’s Total Exposure to Persistent Pesticides and Other Persistent Organic Pollutants (CTEPP) study [12].

Presently, we are unaware of any published data on the temporal variability of urinary PCP concentrations in preschool children. In addition, we are unaware of any published articles that have examined the effect of any sociodemographic or lifestyle factor on urinary PCP concentrations in young children. In this current work, we have conducted a further analyses of the CTEPP data involving preschool children from the OH component of the study. Our objectives were to examine the associations between selected sociodemographic or lifestyle factors and urinary PCP levels in 115 preschool children over a 48-hour monitoring period and to assess the 48-h variability of PCP concentrations in an available subset of 15 of these children.

## 2. Materials and Methods

### 2.1. Study Cohort

In the CTEPP study, we originally investigated the aggregate exposures of 257 preschool children, ages 2–5 years old, and their adult caregivers to over 40 chemicals that were commonly used or found in their everyday environments. An in-depth description of the study design and sampling methodology is described in Wilson *et al.* [19]. Briefly in OH, the study cohort consisted of 127 preschool children and their 127 adult caregivers (usually a parent). In 2001, field sampling activities were performed at 16 daycare centers and 127 homes of study children in six Ohio counties (Cuyahoga, Defiance, Fayette, Franklin, Hamilton, and Licking). About one-half of the children attended daycare (daycare group) during the day while the other half stayed at home (home group) with their adult caregivers. Adult caregivers (parents and daycare teachers) collected spot urine samples from their children and filled out study questionnaires and diaries over a 48-hour monitoring period.

For this present work, we used the subset of 127 preschool children that participated in the OH component of the CTEPP study. We excluded 12 out of 127 children from this dataset because they had missing questionnaire data or diary data. The final dataset consisted of a total of 115 preschool children.

### 2.2. Protection of Human Subjects

The CTEPP study is classified as an observational exposure measurements study as defined in 40 Code of the Federal Regulations, under section 26.402 [20]. The study protocol and procedures to acquire the informed consent of the adult caregivers (parents) and the assent of their children were approved by an independent institutional review board and followed all applicable requirements of the Common Rule (Subpart D) regarding additional protections of a potential sensitive population (children) [20]. The parents also signed an informed consent form prior to their children or themselves participating in this study. In addition, the participants were assigned a study identification number in the publically accessible CTEPP database (http://www.epa.gov/heds/study_75973.html), so their personal information was not identifiable.

### 2.3. Collection of Questionnaires and Diaries

Adult caregivers filled out several different types of hardcopy questionnaires and diaries at home or at daycare during the 48-h monitoring period. The questionnaires and diaries were used to record specific kinds of information and data about the study children including demographics (*i.e.*, age, gender, family income status, and urbanicity), household characteristics, pesticide-use, pet ownership (*i.e.*, dogs or cats), food habits, and activity patterns (e.g., time spent outside).

### 2.4. Collection of Spot Urine Samples

Spot urine samples (up to six) were collected from each child by their adult caregiver at home or at daycare over the 48-h monitoring period. For the home group of children, spot urine samples were collected by their parents in the morning, after lunch, and before bedtime each sampling day. For the daycare group of children, spot urine samples were collected in the morning and before bedtime by their parents each sampling day. In addition, spot urine samples were collected after lunch by classroom teachers each sampling day. The children’s urine samples were collected by inserting a plastic urine bonnet under the toilet seat prior to urination. After urination, the adult caregiver transfered the child’s spot urine sample into a 120 mL plastic container with lid. The urine samples were kept at reduced temperatures in provided coolers with blue ice. At the end of the 48-h monitoring period, field technicians picked up and transported the urine samples by vans to the Battelle laboratory in Columbus, OH, USA. Samples were stored in laboratory freezers at <−20 °C until chemical analyses.

### 2.5. Chemical Analysis of Spot Urine Samples

Detailed information on the preparation, extraction, and analysis of urine samples can be found in Morgan *et al.* [8]. Briefly, the home group of children had their spot urine samples over the 48-h monitoring period pooled into one sample per child. For the daycare group of children, their spot urine samples over the 48-h monitoring period were pooled separately per child into one sample at daycare and into one sample at home. The exception was for 15 children (five in the daycare group and 10 in the home group) that had a recent pesticide application (<7 days) at their homes. Spot urine samples for these children were not pooled, and each sample was analyzed separately.

Each pooled or non-pooled urine sample (1 mL) was hydrolyzed with 100 µL of hydrochloric acid, heated in an oven for one hour at 80 °C, and then 1 mL of 20% sodium chloride and 1 mL of chlorobutane were added to the vial. The extracts were centrifuged, silylated with 100 µL of N-(*tert*-butyldimethylsilyl)-N-methyltrifluoroacetamide, and transferred to a GC vial. The extracts were quantified for levels of total PCP (free PCP and PCP-glucuronide) using a gas chromatography/mass selective detector (Hewlett-Packard 6890/5973A, Agilent Technologies, Golden, CO, USA) in the selected ion-monitoring mode. The limit of quantification (LOQ) for PCP was estimated by using the lowest calibration standard (2 ng/mL) with a signal to noise ratio above two. The estimated LOQ was 0.4 ng/mL for PCP. The limit of detection (LOD) was estimated at one-half the LOQ (0.2 ng/mL) for PCP.

The levels of creatinine were also measured in each child’s pooled urine sample as described in Morgan *et al.* [8]. Briefly, a 10 mL aliquot was taken from each thawed sample and placed into a cryovial with lid at the Battelle laboratory. The urine aliquots were shipped in coolers with dry ice overnight to the Clinical Laboratory, Ohio State University in Columbus, OH, USA. The urine aliquots were quantified for the levels of creatinine using the Jaffe picric colormetric method. Non-pooled urine samples were not analyzed for creatinine concentrations as they generally lacked a sufficient volume for this analysis.

### 2.6. Quality Assurance Procedures

Quality control samples including field and laboratory blanks, matrix spikes, and duplicate samples (field and analytical) were collected in the CTEPP study [8]. All field and laboratory blanks were below the LOD in urine. Matrix spike recoveries ranged from 71%–113% for PCP in urine, except for one sample (64%). Relative percent differences between duplicate field samples (aliquots of the same sample) or duplicate analytical samples (aliquots of the same sample extract) were less than 13% in urine.

### 2.7. Statistical Analyses of Study Data

For PCP, urine measurement values below the LOD were replaced by the LOD divided by the square root of two [21]. For the home group of children, we used the PCP concentration value of each child’s pooled urine sample measurements. For the daycare group of children, we used the mean PCP concentration value of each child’s pooled urine sample measurements that were collected at daycare and at home. In addition, we used the mean PCP concentration value of the non-pooled urine sample measurements for each of the 15 children that had a recent, residential pesticide application. Summary statistics (JMP version 11.1, SAS, Cary, NC, USA) including sample size, frequency of detection, minimum, mean and standard deviation, percentiles (25th, 50th, 75th, and 95th), and maximum were computed for the levels of PCP in the urine of children as unadjusted (ng/mL) and creatinine-adjusted (ng/mg) values.

The intraclass correlation coefficient (ICC) was estimated using PCP measurements of the non-pooled urine samples for the 15 children using a one-way random effects model in SAS 9.4 using PROC MIXED (SAS). The ICC is defined as the ratio of the between-subject variance divided by the total variance. ICC values can range from 0 and 1. An ICC value closer to 1 indicates high reliability, and an ICC value closer to 0 indicates low reliability. An ICC value of ≥0.8 would imply that a single spot urine measurement accurately represents the true mean value over the monitoring period [22]. In addition, we calculated the number of random spot urine samples per child that would be required to obtain a reliable estimate (ICC = 0.80) over the 48-h monitoring period based on the following equation [22]:

m = (p_r,m_(1 – p_r_))/(p_r_(1 – p_r,m_))

where m equals the number of random spot urine measurements per child needed to rank subjects correctly within a population with a defined reliability of the mean (p_r,m_) of 0.80 and p_r_ is the ICC.

The following steps were performed before the multiple regression model was constructed. The distribution of the children’s urinary PCP concentrations was first tested for normality (Shapiro-Wilk test) in GraphPad Prism 5.04 (GraphPad Software, San Diego, CA, USA) and found to be non-normal. To normalize this distribution, we log-transformed (ln) the concentrations of PCP in each urine sample. Then, we selected sociodemographic factors or lifestyle factors from the CTEPP study questionnaires and diaries that have been commonly used in the literature to assess children’s exposures to pesticides. Then in GraphPad Prism 5.04, we used an unpaired *t*-test (*i.e.*, two groups) or an analysis of variance (ANOVA) to assess the bivariate associations between the children’s ln levels of PCP and selected sociodemographic factors (*i.e.*, age group, sex, urbanicity, family income status, site location, and sampling season) and lifestyle factors (*i.e.*, time spent outdoors, age of home, shoe removal before entering home, and pet ownership). In addition, in a separate analysis for the home group of children (n = 66) only, we examined the bivariate associations between their ln urinary PCP levels and selected food frequency consumption categories (*i.e.*, fruits, vegetables, grains, meats, dairy, and snacks). To calculate the food frequency consumption data, we counted how often each child consumed 74 different food items recorded in a food diary over the 48-h monitoring period based on a modified “normal” food habits diary used in Morgan and Jones [23]. Foods that were rarely eaten (e.g., deer meat, zucchini, olives, and Jell-O) were excluded from this list of food items. Then, we placed each child’s consumed food items into the six food consumption categories. One additional child was removed from this analysis as they had incomplete 48-hour food consumption data. Due to excessive missing data on the actual foods consumed at daycare, the daycare group of children were not included in the above analysis.

A multiple regression model was constructed to further evaluate the ln levels of PCP of the children (dependent variable) and sociodemographic and lifestyle factors (independent variables) that had *p*-values of ≤0.100 in our above bivariate analyses. Creatinine concentrations (logged) were also included in this model as an independent variable to adjust for dilutions in urine volumes [24]. We performed our multiple regression analysis using a sequential, step-wise backward elimination process in SAS 9.4 using PROC GLM.

## 3. Results

### 3.1. Urinary Concentrations of PCP

Table 1 provides the summary statistics for the unadjusted (ng/mL) and creatinine-adjusted (ng/mg) levels of urinary PCP over a 48-hour period for all children and by group (home and daycare). PCP was detected in 99% of unadjusted urine samples across all 115 children. The median PCP level for all children was 0.8 ng/mL (range ≤0.2–23.8 ng/mL).

**Table 1:** Urinary levels of PCP in children over a 48-h monitoring period **^a^**.

Descriptive Statistic	All Children	Home Group	Daycare Group
**ng/mL**			
Number	115	67	48
% ^**b**^	99	99	100
Mean ± SD	1.3 ± 2.3	1.6 ± 2.9	0.99 ± 0.65
Minimum	<0.2	<0.2	0.23
25th	0.60	0.64	0.49
50th	0.83	0.91	0.77
75th	1.4	1.4	1.5
95th	3.3	4.6	2.5
Maximum	23.8	23.8	2.8
IQR ^**c**^	0.82	0.78	0.97
**ng/mg-creatinine ^d^**			
Number	100	57	43
%	99	99	100
Mean ± SD	1.8 ± 2.3	2.1 ± 2.9	1.4 ± 1.0
Minimum	<0.2	<0.2	0.27
25th	0.82	0.96	0.65
50th	1.2	1.3	1.1
75th	1.9	2.0	1.8
95th	5.0	5.6	3.6
Maximum	21.4	21.4	5.0
IQR	1.1	1.0	1.2

**^a^** Unadjusted urinary PCP concentrations were calculated using data for 115 out of 127 children from Wilson *et al.* [12]. ^**b**^ Percentage of urine samples at or above the limit of detection; ^**c**^ Interquartile range; ^**d**^ Creatinine was not measured in the urine samples of 15 children that had a recent pesticide application; (<7 days) at home as these samples typically had low volumes of urine.

The median levels of urinary PCP were slightly higher for the home group of children (0.91 ng/mL) compared to the daycare group of children (0.77 ng/mL). In addition, the maximum PCP concentration of 23.8 ng/mL occurred for one child in the home group of children. For the creatinine-adjusted values, the children’s median PCP concentrations were 1.2 ng/mg (range ≤0.2–21.4 ng/mg) again slightly greater for the home group of children (1.3 ng/mg) compared to the daycare group of children (1.1 ng/mg).

### 3.2. Variability of Urinary PCP Concentrations over a 48-h Period

The results from the random-effects model showed a between-subject variance component estimate of 0.34 and a within-subject variance component estimate of 0.46, resulting in an ICC estimate of 0.42. This ICC value indicated a fairly low level of reliability for a child’s spot urine sample over the 48-h monitoring period. To obtain a reliable estimate (ICC = 0.80) that would allow meaningful exposure classification, the results indicate that at least five spot urine measurements would be needed per child over the 48-h monitoring period.

### 3.3. Predictors of Urinary PCP Concentrations

Table 2 provides the bivariate associations between selected sociodemographic or lifestyle factors and urinary PCP concentrations in the preschool children. The results show that the urinary PCP levels (log-transformed) were statistically significantly higher (*p* = 0.041) in children living in rural counties (GM = 1.3 ng/mL) compared to those in urban counties (GM = 0.87 ng/mL). The children’s urinary PCP concentrations were also statistically significantly different (*p* = 0.027) across the three sampling seasons, with the highest levels occurring in the summertime (GM = 1.1 ng/mL). In addition, urinary levels of PCP were significantly higher (*p* = 0.028) in children that spent >2 h *vs.* ≤ 2 h outside per sampling day. Urinary PCP levels were also statistically significantly higher (*p* = 0.0004) in children that lived in older homes (>15 years old) compared to newer homes (≤15 years). Lastly, urinary PCP concentrations were statistically significantly greater (*p* = 0.049) in children that live with a pet dog/cat (GM = 1.1 ng/mL) compared to those without a dog/cat (GM = 0.80 ng/mL).

Table 3 presents the bivariate associations between selected food frequency consumption categories and ln urinary PCP levels in the home group of children. No significant association (*p* > 0.05) was observed between any food consumption category (fruits, vegetables, meats, dairy, grains, and snacks) and the children’s urinary PCP concentrations by intake group.

The results of our final reduced regression model of sociodemographic or lifestyle factors influencing the ln PCP concentrations for the preschool children are provided in Table 4. The results showed that age of home (*p* = 0.012) and ln(creatinine levels) (*p* = 0.004) were significant predictors and sampling season, time spent outside, and owning a pet dog or cat were marginally statistically significant predictors of ln(urinary PCP level), together explaining 29% of the variability in PCP concentrations in the children’s urine samples. In addition, the results showed that age of home, sampling season, time spent outside, and pet ownership collectively explained the majority (19%) of the variability of PCP in the children’s urine samples. In particular, urinary PCP levels were significantly (*p* = 0.012) greater in children that lived in older homes (>15 years old) compared to newer homes (≤15 years). The children also had marginally statistically significant (*p* = 0.066) different concentrations of PCP across the three sampling seasons with summertime having the highest biomarker levels. In addition, the children had marginally statistically significant (*p* = 0.077) higher urinary levels of PCP for those that spent >2 h *vs.* ≤2 h outside each sampling day. Lastly, the children had marginally statistically significant (*p* = 0.068) greater PCP levels for those that owned a pet (dog or cat) compared to those that did not own a pet.

**Table 2:** Urinary PCP levels (ng/mL) in all children by sociodemographic or lifestyle factor.

Variable	N ^a^	% ^b^	GM ^c^	95% CL ^d^	*p*-value
**Sociodemographic factor**					
Age group					
<48 months	54	47	0.90	0.74–1.1	0.874
≥48 months	61	53	0.92	0.76–1.1	
Sex					
Male	58	50	0.93	0.78–1.1	0.725
Female	57	50	0.89	0.72–1.1	
Urbanicity (county-level)					**0.041 ^g^**
Urban	101	88	0.87	0.75–1.0	
Rural	14	12	1.3	0.96–1.9	
Family income status ^**e**^					
Low-income	36	35	0.89	0.71–1.1	0.606
Middle/high-income	67	65	0.97	0.79–1.2	
Site location					
Home	67	58	1.0	0.83–1.2	0.127
Daycare	48	42	0.81	0.67–0.97	
Sampling season ^**f**^					
Spring	41	36	0.73	0.60–0.89	**0.027**
Summer	57	49	1.1	0.93–1.3	
Fall	17	15	0.84	0.48–1.5	
**Lifestyle factor**					
Time spend outdoors per sampling day					
≤2 h	58	50	0.79	0.66–0.94	**0.028**
>2 h	57	50	1.1	0.87–1.3	
Age of home					
≤15 years	31	27	0.62	0.49–0.78	**0.0004**
>15 years	84	73	1.1	0.91–1.2	
Remove shoes before entering home					
Yes	44	38	0.87	0.70–1.1	0.585
No	71	62	0.94	0.79–1.1	
Own a pet (dog or cat)					
Yes	56	49	1.1	0.83–1.3	**0.049**
No	59	51	0.80	0.69–0.92	

Notes: **^a^** Number of children; ^**b**^ Percentage of children; ^**c**^ Geometric mean; ^**d**^ Confidence limits; ^**e**^ Missing data on income status for 12 children; ^**f**^ Field sampling activities were performed between April 2001 and November 2001; ^**g**^ Statistically significant associations (*p* < 0.05) are in bold text.

**Table 3:** Urinary PCP levels (ng/mL) in the home group of children by food consumption category **^a^**.

Food Category	N ^a,b^	% ^c^	GM ^d^	95% CL ^e^	*p*-value
Fruits					0.747
<2 times	34	52	0.96	0.76–1.3	
≥2 times	32	48	1.0	0.82–1.3	
Vegetables					0.182
<2 times	27	41	0.85	0.63–1.1	
≥2 times	39	59	1.1	0.86–1.4	
Meats					0.160
≤2 times	36	55	0.87	0.68–1.1	
>2 times	30	45	1.2	0.87–1.5	
Dairy					0.306
≤2 times	35	53	1.1	0.83–1.4	
>2 times	31	47	0.89	0.68–1.2	
Grains					0.879
≤4 times	32	48	1.0	0.82–1.2	
>4 times	34	52	0.98	0.71–1.4	
Snacks ^**f**^					0.543
≤4 times	26	39	1.1	0.79–1.5	
>4 times	40	61	0.95	0.74–1.2	

**^a^** Number of children; ^**b**^ One child was excluded from this analysis as they has incomplete food consumption data over the 48-h monitoring period; ^**c**^ Percentage of children; ^**d**^ Geometric mean; ^**e**^ Confidence limits; **^f^** The snacks category include such items as candies, cakes, cookies, popcorn, chips, and crackers.

**Table 4:** Final reduced regression model of factors influencing ln urinary PCP levels in children **^a,b^**.

Factors	Type ^c^	β Coefficient	SE ^d^	*p*-value
Sampling season	SD			0.066
Spring		−0.228	0.228	
Summer		0.138	0.215	
Fall		0 (ref.)	----	
Time spent outdoors	LS			0.077
>2 h		0.266	0.149	
<2 h		0 (ref.)	----	
Age of home	LS			**0.012 ^f^**
>15 years		0.438	0.170	
<15 years		0 (ref.)	----	
Own a pet dog or cat	LS			0.068
Yes		0.257	0.139	
No		0 (ref.)	----	
Creatinine level ^**e**^	----	0.487	0.164	**0.004**

**^a^** A total of 100 children were used in this model; ^**b**^ The r^2^ = 0.29; ^**c**^ Sociodemographic (SD) or lifestyle (LS) factor; **^d^** Standard error; **^e^** Continuous variable (log-transformed); units are mg/dL; ^**f**^ Statistically significant variables (*p* < 0.05) are in bold text.

## 4. Discussion

A limited number of studies (Table 5) have been published worldwide on children’s exposures to PCP using urinary biomonitoring data [11,12,18,25,26,27,28,29,30]. Of these studies, urinary PCP concentration data only exist for younger children (<6 years) in the U.S. Hill *et al.* [18] reported median PCP levels of 14 ng/mL for 197 children, ages 2–6 years old, in the 1980’s. In another study conducted by Wilson *et al.* [11], they had much lower mean urinary PCP concentrations of 0.3 ng/mL for nine preschool children, 2–5 years old, in NC in 1997. Our CTEPP study results are more similar to Wilson *et al.* [11] having mean urinary PCP levels of 1.3 ± 2.3 ng/mL (median = 0.8 ng/mL) for 115 OH preschool children in 2001. In addition in comparison to our study, the 2003–2004 U.S. National Health and Nutrition Examination Survey (NHANES), a population-based study, had lower median PCP levels (<0.5 ng/mL) for older children (6–11 years old) [25]. However at the 95th percentile, the urinary PCP levels were higher for the NHANES children (5.7 ng/mL) compared to the CTEPP children (3.3 ng/mL) (Table 5). Together, these studies have confirmed that U.S. children are still being exposed to PCP after it was banned in 1987 for almost all uses, except for wood preservation in limited applications (e.g., utility poles) [3]. However based on these limited data, it remains unclear whether children’s exposures to PCP in their everyday environments have substantially declined over time in the U.S. after the U.S. EPA’s regulatory actions in the late 1980’s. As PCP is classified as a probable human carcinogen by the U.S. EPA [3] and as a possible human carcinogen (Group 2B) by the International Agency for Research on Cancer [31], more data are needed on the important sources and routes of young children’s exposures to PCP in their everyday environments in the U.S. and globally.

**Table 5:** Urinary PCP levels (ng/mL) in young children from published studies worldwide **^a,b^**.

Country	Location	Year	N ^c^	Age (Years)	Median	95th	Maximum	Reference
Germany	National (GerES II)	1990–1992	695	6–14	4.6	14.9	26.5	Seifert *et al.* [26]
Germany	National (GerEs IV)	2003–2006	462	6–14	<0.6	1.6	----	Schultz *et al.* [27]
USA	National (NHANES) ^**d**^	2003–2004	290	6–11	<0.5	5.7	----	CDC [25]
USA	AK, USA	1980’s ^**e**^	197	2–6	14	110	240	Hill *et al.* [18]
USA	NC, USA	1997	9	2–5	0.3 ^**f**^	----	0.7	Wilson *et al.* [11]
USA	NC, USA	2000–2001	128	2–5	0.4	1.9	3.5	Wilson *et al.* [12]
USA	OH, USA	2001	115	2–5	0.8	3.3	23.8	Current study ^**g**^

**^a^** All of these studies measured for total PCP in urine; **^b^** Urinary PCP levels for only children are not listed in Thompson and Treble [28,29]; In these two studies, summary data were reported for all subjects (ages 4–62 years old) from Saskatchewan, Canada in 1992 and 1995; **^c^** Number of children; **^d^** The 1999–2002 NHANES data are not provided as they were withdrawn by the CDC because of “unacceptable calibration bias” [30]; ^**e**^ Estimated date; ^**f**^ Mean value (no median value provided); ^**g**^ Values were calculated using data for 115 out of 128 children from Wilson *et al.* [12].

We believe this is the first study to publish data on the short-term variability of PCP in the urine of preschool children. Our results showed fairly low reliability (ICC = 0.42) of repeated PCP measurements in the urine samples of 15 CTEPP OH children over a 48-hour monitoring period. This information suggested that these children were likely being intermittently exposed to PCP from various sources and pathways in their daily environments. Our results also indicated that several spot urine measurements were needed over a day to provide a reliable estimate of preschool children’s exposure to PCP in these settings. However due to the small sample size of children in our study, additional research is needed to confirm our above findings on the (short-term) variability of PCP in the urine of children.

Assuming steady-state conditions of PCP, the CTEPP children’s estimated maximum intake dose (0.53 µg/kg-day) was approximately nine times lower than the established oral reference dose (RfD) of 5 µg/kg-day listed by the U.S. EPA’s Integrated Risk Information System [6]. We calculated the children’s maximum intake dose of PCP by multiplying the highest urinary PCP level for a study child (23.8 ng/mL) by a daily urine excretion rate (22.4 mL/kg body weight) of young children [11,32,33]. Based on the 48-h urine concentration data, this information suggests that the CTEPP children’s exposures to PCP were low as compared to the oral RfD.

At the moment, it is unclear whether the levels of PCP measured in the CTEPP children’s 48-h urine samples reflect more recent or past environmental exposures. Only a few studies have been conducted that have examined the toxicokinetics (*i.e.*, half-life) of PCP in human volunteers; however, these studies have produced conflicting results [1,16,17]. Braun *et al.* [16] reported that four, male adults (fasted 8-h) administered a single, oral dose of 0.1 mg Na-PCP/kg body weight dissolved in water had an average urinary elimination half-lives of ~30 and 13 h for free PCP and PCP-glucuronide, respectively. In a later study conducted by Uhl *et al.* [17], three adult males (non-fasted) given a single oral dose of technical grade PCP at 3.9, 4.5, or 9.0 mg dissolved in 40% ethanol had a much longer average urinary elimination half-life of about 20 days for total PCP. It appears that the vastly different urinary elimination half-lives of PCP between the two studies are likely due to study design differences [1]. More research is necessary on quantifying the half-life of PCP in humans to elucidate its persistence or not in the body.

There is currently conflicting evidence on whether dietary ingestion is a major exposure route of children to PCP in the U.S. [12,13,15,34]. Hattemer-Frey and Travis [13] reported that the consumption of vegetables, fruits, and grains contributed to almost all (99.9%) of the nonoccupational exposures of humans to PCP. However in the US Food and Drug Administration’s Total Diet Study (TDS) (1991–2004), PCP residues were not found in any sampled fruit, vegetables, or grains, purchased from supermarkets in four different geographical regions of the country [34]. In the TDS study, PCP residues were only reported in one sample each of baked/cured ham (0.02 µg/g) and oven-roasted chicken breast (0.01 µg/g) from supermarkets in four different geographical regions of the country [34]. More recently, Wilson *et al.* [15] showed that dietary ingestion of composited food samples contributed to approximately 45% of the aggregate potential doses of 101 preschool children to PCP at their homes in NC over a three year period (2003–2005). In contrast, our previous research [12] showed that dietary ingestion of composited food samples over a 48-h period was a minor route of the CTEPP children’s exposures to PCP in NC and OH in 2000–2001. In support of this finding, in our current bivariate analysis we also did not find any significant associations between the home group of children’s ln(urinary PCP) levels and any food frequency consumption category (fruits, vegetables, meats, dairy, grains, and snacks) by intake group over the 48-h monitoring period. This research suggests that there is likely substantial temporal variability in children’s dietary exposures to PCP, and more information is needed on the specific foods or food categories that contribute to their exposures.

We are also unaware of published research that has reported the influence of any sociodemographic or lifestyle factor on urinary PCP concentrations in young children. Our study results showed that age of home and ln(creatinine levels) were significant predictors and sampling season, time spent outside, and owning a pet dog or cat were marginally statistically significant predictors of ln(urinary PCP level), collectively explaining 29% of the variability of PCP in the children’s urine samples. An important result was that urinary PCP levels were significantly (*p* = 0.012) greater in CTEPP OH children that lived in older homes (>15 years old) compared to newer homes (≤15 years). Before 1987, the semivolatile PCP was commonly used in pressure-treated lumber and in paints, stains, and sealants to protect wood from insect and fungal damage in dwellings (*i.e.*, residences, schools, and gymnasiums) [2,5,35]. Our results are supported by research conducted by Colt *et al.* [36] showing that age of home was a significant predictor of organochlorine concentrations (including PCP) in dust samples collected from 1046 homes in California, Iowa, Michigan, and Washington in 1998–2000. The authors found that the lowest PCP levels occurred in dust samples from residences built after 1980 [36]. Another interesting study result was that the CTEPP OH children had marginally statistically significant (*p* = 0.066) different urinary concentrations of PCP among the three sampling seasons (spring, summer, and fall) with summer having the highest biomarker levels. This is in agreement with Thompson and Treble [29] that also reported seasonal differences in the urinary levels of PCP in the fall of 1992 (median = 1.3 ng/mL) compared to the winter of 1995 (0.5 ng/mL) for Canadians ages 4–62 years old in Saskatchewan. In addition, research conducted by Waite *et al.* [37] showed that ambient PCP levels were substantially higher in the summer months (July to August) compared to the winter months (November to January) at five sampling sites in North America (Canada) in 1995 and 1996. In addition, we found that the CTEPP OH children had marginally statistically significant (*p* = 0.077) higher urinary levels of PCP that spent >2 h compared to ≤2 h outside each sampling day. Interestingly in the CTEPP study, median levels of PCP were 0.43 ng/m^3^ and 0.22 ng/m^3^ in the outdoor air samples at the OH children’s homes and daycare centers, respectively—which were the highest outdoor air levels reported among all measured chemicals (except for di-*n*-butylphthalate) [8]). Perhaps in outdoor settings, these children were being exposed to measureable levels of PCP directly by air and/or indirectly following volatilization of it from treated lumber or painted/stained wood surfaces [5]. Lastly, our study results showed that CTEPP OH children had marginally statistically significant (*p* = 0.068) higher urinary levels of PCP for those that owned a pet (dog or cat) compared to those that did not own a pet. In support of our finding, Lu *et al.* [38] also found significantly (*p* = 0.04) greater levels of pesticide (dimethyl diakylphosphate) metabolites in 110 Seattle, WA children, ages 2–5 years old, that had a household pet (cat or dog) compared to those that did not have a household pet. This information suggests that pets may be tracking in outdoor PCP residues onto their paws and fur into homes and/or directly exposing children through personal contacts (*i.e.*, petting) [39]. Our above research findings suggests that certain sociodemographic factors (*i.e.*, sampling season) or lifestyle factors (*i.e.*, age of home, time spent outdoors, and pet ownership) can substantially influence the variability of PCP concentrations in preschool children. In addition, these factors suggests a linkage between young children’s exposures to PCP mainly through the inhalation of air and urinary concentrations of PCP at their homes and daycare centers. Lastly as we have accounted for only 29% of the variability of PCP, this information suggest that other unknown factors are likely substantially contributing to the short-term variability of PCP in the CTEPP children’s urine samples.

## 5. Conclusions

In conclusion, the urinary biomonitoring data confirmed that almost all of these CTEPP OH children were exposed to PCP in their daily environments. The variability in the children’s urinary PCP measurements over a 48-h period suggested that several spot urine samples are needed over a day to adequately assess short-term exposures to PCP in these settings. In addition, we identified specific factors (*i.e.*, age of residence, sampling season, time spent outside, and pet ownership) that increased these children’s exposures to PCP at their homes and daycare centers in OH.
